# Safety and efficacy of the ShangRing for early infant male circumcision in the routine clinical setting

**DOI:** 10.1136/bmjgh-2024-017903

**Published:** 2025-09-25

**Authors:** Lina Posada Calderon, Spyridon P Basourakos, Karla V Ballman, Kaylee Ho, Mark A Barone, Quentin Awori, Daniel Ouma, Jairus Oketch, Alice Christensen, Augustino Hellar, Maende Makokha, Alphonce Isangu, Robert Salim, Jackson Lija, Ronald Gray, Stephen Kiboneka, Aggrey Anok, Godfrey Kigozi, Regina Nakabuye, Charles Ddamulira, Silas Odiya, Marc Goldstein, Philip S Li, Richard K Lee

**Affiliations:** 1 Department of Urology, Weill Cornell Medicine, New York-Presbyterian Hospital, New York, New York, USA; 2 Mayo Clinic Minnesota Department of Urology, Rochester, Minnesota, USA; 3 Department of Population Health Sciences, Mayo Clinic College of Medicine and Science, Rochester, Minnesota, USA; 4 Department of Population Health Sciences, Weill Cornell Medicine, New York, New York, USA; 5 Population Council Center for Biomedical Research, New York, New York, USA; 6 Bill & Melinda Gates Foundation, Seattle, Washington, USA; 7 Population Council Kenya, Nairobi, Kenya; 8 Homa Bay Teaching and Referral Hospital, Homa Bay, Kenya; 9 Jhpiego Tanzania, Dar es Salaam, Tanzania, United Republic of; 10 Ministry of Health, Dar es Salaam, Tanzania, United Republic of; 11 Epidemiology, Johns Hopkins University Bloomberg School of Public Health, Baltimore, Maryland, USA; 12 Rakai Health Sciences Program, Kalisizo, Uganda

**Keywords:** HIV, Prevention strategies, Health policy, Urology, Kenya

## Abstract

**Introduction:**

Male circumcision (MC) has been shown to decrease transmission of HIV and is a critical strategy to decrease its incidence in countries with high rates of heterosexually transmitted HIV. Early infant MC (EIMC), with single-use MC devices, could increase rates of MC in Sub-Saharan Africa. Our objective was to determine the safety, efficacy and satisfaction of ShangRing EIMC in resource-constrained environments.

**Methods:**

Healthy male infants aged 1–60 days underwent ShangRing EIMC performed by non-physician clinicians in 14 sites in Kenya, Tanzania and Uganda. The primary outcomes were the safety and efficacy of ShangRing EIMC. Secondary outcomes were pain related to the procedure, wound healing and parental satisfaction. Male infants were assessed at discharge and on postoperative days 7 and 28 for wound healing and complications. This trial is registered with Clinical.Trials.gov, NCT03338699, and is complete.

**Results:**

From the 1697 participants enrolled from February to November 2020, 1691 (99.6%) underwent ShangRing EIMC. Median duration of the procedure was 10 min (IQR 6–13). The mean Neonatal Infant Pain Scale 20 min postprocedure was 0.3 (SD 0.8), indicating no pain. At the time of discharge, 1679 (99.0%) participants had a normal-appearing wound and 1574 (93.1%) demonstrated complete wound healing at 28 days. A total of 17 (1.0%) AEs were reported, most related to bleeding. A total of 1635 (99.1%) of parents were satisfied with the appearance of the circumcised penis at 28 days.

**Conclusion:**

ShangRing EIMC is a simple and successful MC method that non-physician clinicians can perform safely, with low AE rate, satisfactory wound healing and high satisfaction rate among parents. This indicates that the ShangRing EIMC can be considered a long-term strategic approach to accelerate the scale-up of MC in Sub-Saharan Africa.

**Trial registration number:**

NCT03338699.

**WHAT IS ALREADY KNOWN ON THIS TOPIC**

**WHAT THIS STUDY ADDS**

- In this multi-institutional field study in Kenya, Tanzania and Uganda, we show the performance of Shang Ring EIMC performed by non-physician clinicians in the real-world, resource-limited setting. The ShangRing EIMC showed to be safe and efficient, with satisfactory wound healing and high satisfaction rate among parents.

**HOW THIS STUDY MIGHT AFFECT RESEARCH, PRACTICE OR POLICY**

- Encouraging real-world implementation data for the ShangRing in the EIMC setting should result in increased access to the device and circumcision. Ease of use and efficiency when performed by non-physician clinicians should motivate policy makers in resource-limited settings to adopt this technique as a method to accelerate the scale-up of voluntary medical male circumcision in Sub-Saharan Africa.

## Introduction

 Circumcision is known to lower the risk of acquiring HIV, as demonstrated by three randomised controlled trials revealing a 50% to 60% reduction in HIV incidence among men.^1^,^3^ Since 2007, the WHO and the Joint United Nations Programme on HIV/AIDS (UNAIDS) have recommended voluntary medical male circumcision (VMMC) as a critical strategy to prevent and decrease the incidence of HIV in countries with high rates of heterosexually transmitted HIV.^4^ As of 2021, approximately 21 million VMMC have been performed in Sub-Saharan Africa, and it is estimated that one HIV transmission is prevented for every 80 circumcisions performed.^5 6^

Traditional MC has a heterogeneous distribution and occurs at varying ages according to specific cultural or religious practices.^7^ Device-assisted MC offers a safe, simple and cost-effective alternative to VMMC.^4 8^ The ShangRing is currently the only WHO-prequalified circumcision device approved for VMMC in Africa. Its implementation has primarily been driven by the President’s Emergency Plan for AIDS Relief efforts and national VMMC programmes, with most experience concentrated in Kenya, Uganda, Malawi, Tanzania and Zambia, primarily among adults and adolescents. However, these efforts alone have not been sufficient to achieve the projected goal of 90% access to VMMC.^4 8^ This has led to increased interest in device-assisted early infant MC (EIMC) as a means of reaching this target.

EIMC, though not as thoroughly examined as VMMC, presents a viable approach that could aid in achieving a higher circumcision rate. Studies have indicated that EIMC is safe, can offer protection before starting sexual activity and removes concern for resuming sexual activity during recovery. Although it has been shown to be more cost-effective at reducing lifetime HIV risks,^9^,^11^ it continues to be less accepted than VMMC primarily because of lack of knowledge, cultural attitude and assumptions.^12^ Ultimately, while adolescent and adult circumcision programmes have successfully increased coverage in some countries, maintaining these long-term results requires a task shift toward routine circumcision at younger ages to ensure each new generation is circumcised without requiring large-scale catch-up campaigns.

In various clinical trials, the ShangRing MC with adults, adolescents and early infants has been proven to be safe and to have shorter operative times, lower intraprocedural pain score, less blood loss, fewer adverse events (AEs) and higher satisfaction with penile appearance compared with conventional circumcision.^13^,^16^ Results showed promising potential to scale up EIMC in Sub-Saharan Africa.^17^ To the best of our knowledge, this is the first large cohort that has studied the use of the ShangRing for EIMC by midlevel medical providers in routine clinical settings.

## Materials and methods

Following the WHO Framework for Clinical Evaluation of Devices for Male Circumcision, the clinical outcomes of the ShangRing were studied in a non-comparative field study in routine clinical settings, with circumcision performed by trained midlevel non-physician medical providers.^18^ Expecting parents of newborn males or legally authorised representatives (LARs) at proposed sites in Uganda, Tanzania and Kenya were screened. Enrolling sites in Uganda were in clinics in Kakuuto, Kalisizo, Lyantonde, Masaka and Rakai; in Tanzania were Frelimo Hospital, Ipogoro Health Center, Ngome Health Center, Iringa Regional Hospital, Mafinga District Hospital and Ilula Hospital; and in Kenya were in Homa Bay, Jaramogi Oginga Odinga Teaching & Referral Hospital and Nyanza Reproductive Health Society. The information on the risks and benefits of EIMC using the Uganda, Tanzania and Kenya Ministry of Health and WHO/JHPIEGO guidelines was provided to parents or LARs aged 18 years or older. Those willing to participate were asked to provide written informed consent for screening and enrolment. Our research adhered to the set of principles outlined by the Declaration of Helsinki, and the Institutional Review Board approved this study at our institution (Weill Cornell Medicine IRB #1611017762) as well as in each country in Africa where it was performed. This trial is registered with Clinical.Trials.gov (NCT03338699) as the phase 2 of the study, fulfilling the requirements for the WHO prequalification of MC devices.

### Study participants and procedures

Healthy male infants aged 1 to 60 days, with a gestational age ≥37 weeks and birth weight ≥2.5 kg, were identified and screened at all study sites. Participants who followed inclusion and exclusion criteria as previously described in detail in our randomised controlled trial were deemed eligible to participate, and parent(s)/LARs were consented.^16^ Appropriate referrals were made if parents were interested in EIMC, but their children did not meet inclusion criteria for the study. Participants who were enrolled were not involved in research design or analysis.

All infants received the ShangRing EIMC performed by trained midlevel non-physician medical providers who completed a hands-on training course, following the same protocol as previously described.^16 19 20^ Topical anaesthesia (2.5% lidocaine and 2.5% prilocaine mix) was applied to the glans and shaft of the penis, and dwell time was determined by absence of response to noxious stimuli. If needed, providers could use additional injectable local anaesthesia. Dwell time and need for additional anaesthesia were recorded. After EIMC, the wound was dressed, and the infant’s family was instructed that the ShangRing device would either fall on its own or be removed by the healthcare provider at follow-up. Participants were assessed at discharge and on postoperative days 7 and 28, with unscheduled visits made as needed. The ShangRing was allowed to detach spontaneously or was removed at the visit on day 28 if still not detached. Infants were examined for wound healing and complications, and parents were encouraged to return to the healthcare provider at any time if there was any complication, excessive pain or any other problem. Difficulties with the procedures were reported by parents at the time of post-operative visits as needed.

### Outcomes

The primary outcomes were the safety and efficacy of ShangRing EIMC in a routine setting. Secondary outcomes were pain related to the procedure, operative time, time to spontaneous detachment of the ShangRing device, time to complete wound healing, parental satisfaction and AEs. Pain score was assessed using the Neonatal Infant Pain Scale (NIPS), assessed immediately prior to the procedure and 20 min postprocedure. The NIPS is a commonly used acute pain scoring system validated in the neonatal intensive care unit, based on facial expression, crying, breathing patterns, movement of arms and legs and state of arousal.^21^ Parental satisfaction was explored with questionnaires that included what they liked and disliked about the EIMC and the likelihood of recommending it. AEs were classified by severity and if they were related or unrelated to EIMC, based on WHO definitions.^19^ Additionally, following the WHO guidelines framework for MC devices,^18^ device malfunction and need for removal was appropriately reported.

### Statistical analysis and sample size

The WHO recommends at least 500 participants per country for the field studies with clinical assessment.^18^ Continuous data were summarised as mean ± standard deviation (±SD) or median (IQR), and categorical data were summarised as frequency (relative frequency or %). The statistical analysis was performed on the participants that received the ShangRing, and those that did not receive it were excluded from the analysis. Statistical analyses were performed using R V.3.6.1.

### Patient and public involvement

The research question and outcome measures were developed in alignment with the WHO Framework for the clinical evaluation of MC devices. This framework was established to standardise device approval across multiple sub-Saharan countries. The approval process required an initial randomised controlled trial—previously conducted and published by our group—followed by a field study involving 500 participants per country. Patients and the public were not directly involved in the study design, as it was structured according to the framework’s outlined requirements. The findings will be disseminated through peer-reviewed publications, conference presentations and popular media. No formal patient advisors were involved in the study.

## Results

From 1743 screened participants, 1697 were enrolled from February 2020 to November 2020, of which 1691 (99.6%) underwent a ShangRing EIMC procedure. The median age at the time of the procedure was 29 days, with an IQR of 13–45, and the mean weight was 4.31 kg and the SD of 0.95. A total of 1371 (81.1%) had a spontaneous vaginal delivery, and 1657 (98.0%) were delivered at a facility. The most common reasons for parents/LARs to seek EIMC were hygiene in 769 (45.5%) cases and HIV prevention in 718 (42.5%) cases. The remaining 40 (2.4%) participants were lost to follow-up, and in one case, the Shang Ring was removed on the day of surgery because of bleeding (table 1).

**Table 1:** Characteristics of the participants in the ShangRing field study

	ShangRing (n=1691)	Other (n=6)	Total (n=1697)
Age (days), median (IQR)	29 (13–45)	9 (6-14)	29 (13–45)
Weight (kg), mean (SD)	4.31 (±0.95)	3.88 (±0.89)	4.31 (±0.95)
Age of parent/LAR (years), median (IQR)	27.0 (23.0–31.8)	31.0 (27.1–35.1)	27.0 (23.0–31.8)
Mode of delivery, n (%)			
Spontaneous vaginal delivery	1371 (81.1)	5 (83.3)	1376 (81.1)
Caesarean section	319 (18.9%)	1 (16.7)	320 (18.9)
Site of delivery, n (%)			
Facility	1657 (98.0)	6 (100)	1663 (98.0)
Home	33 (2.0)	0 (0)	33 (1.9)
Other	2 (<0.1)	0 (0)	2 (<0.1)
Country, n (%)			
Uganda	550 (32.5)	3 (50.0)	553 (33.0)
Tanzania	599 (35.4)	1 (16.7)	600 (35.4)
Kenya	542 (32.0)	2 (33.3)	544 (32.1)
Reason for parent/LAR seeking circumcision, n (%)			
Hygiene	769 (45.5)	3 (50.0)	772 (45.5)
HIV prevention	718 (42.5)	1 (16.7)	719 (42.4)
Other	113 (6.7)	1 (16.7)	114 (6.7)
Social religious	51 (3.0)	1 (16.7)	52 (3.1)
Medical therapy	40 (2.4)	0 (0)	40 (2.4)
Completed study*	1650 (97.6)	0 (0)	1650 (97.2)

* Attended follow-up through 28 days.

LAR, legally authorised representative.

### Safety and practicality of procedure by midlevel medical provider

The procedure had a median duration of 10 min (IQR 6–13). The performing provider was a nurse in 1000 cases (59.1%) and a clinical/medical officer in 690 cases (40.8%). In six (0.4%) cases, there was a reported difficulty with the ShangRing EIMC procedure by the providers, and almost all were related to small size of the penis (online supplemental table 1). A dorsal slit in the foreskin was required to insert the ring in 1428 (84.4%) participants (table 2).

**Table 2:** Perioperative characteristics

	ShangRing (n=1691)
Procedure duration (minutes), median (IQR)	10 (6–13)
Type of anaesthesia, n (%)	
Topical cream alone	1646 (97.3)
Topical with injectable	44 (2.6)
Anaesthetic dwell time (minutes), mean (SD)	57.5 (±18.6)
Cadre of providers performing circumcision, n (%)	
Nurse	1000 (59.1)
Clinical officer	690 (40.8)
Unknown	1 (<0.1)
Cadre of providers assisting circumcision	
Nurse	1143 (67.6)
Clinical officer	547 (32.3)
Wound status prior to discharge	
Normal	1679 (99.3)
Abnormal (penile skin swelling)	1 (<0.1)
Missing	11 (0.7)
ShangRing size (mm), n (%)	
W (14 mm)	2 (<0.1)
X (13 mm)	102 (6.0)
Y (12 mm)	582 (34.4)
Z (11 mm)	1004 (59.4)
Missing	1 (<0.1)
Need for dorsal slit in the foreskin to insert ring, n (%)	1428 (84.4)
ShangRing still in place at visit day 7	297 (17.6)
Completely healed on visit day	
Day 7	9 (0.5)
Day 28	1574 (93.1)
Unscheduled (days 21–180)	67 (4.0)
Lost to follow-up	41 (2.4)

### Pain assessment

In 1646 (97.3%) cases, only topical cream as a local anaesthetic was used while in 44 (2.6%) participants required supplemental local injectable anaesthesia. The mean anaesthetic dwell time was 57.5 (±18.6) minutes. The pre-procedural mean NIPS score was 2 (SD 2) and at 20 min postoperatively 0.3 (SD 0.8) (table 3).

**Table 3:** Neonatal Infant Pain Scale (NIPS) immediately prior and 20 min after the circumcision

		Prior to circumcision (N=1691)	20 min postcircumcision (N=1691)
Facial expression	Relaxed	871 (51.5%)	1588 (93.9%)
	Grimacing	819 (48.4%)	92 (5.4%)
Cry	No cry	373 (22.1%)	1485 (87.8%)
	Whimper	909 (53.8%)	188 (11.1%)
	Vigorous cry	408 (24.1%)	7 (0.4%)
Breathing pattern	Relaxed	1282 (75.8%)	1666 (98.5%)
	Change in breathing	408 (24.1%)	21 (1.2%)
Arms	Restrained or relaxed	1408 (83.3%)	1582 (93.6%)
	Flexed or extended	282 (16.7%)	98 (5.8%)
Legs	Restrained or relaxed	1452 (85.9%)	1592 (94.1%)
	Flexed or extended	238 (14.1%)	88 (5.2%)
State of arousal	Sleeping or awake	1161 (68.7%)	1628 (96.3%)
	Fussy	529 (31.3%)	52 (3.1%)
Total NIPS score			
Mean (SD)		2 (2)	0.3 (0.8)
Min–max		0–7	0–5

### Time to complete healing

At the time of discharge, 1679 (99.3%) participants had a normal-appearing wound, and a completely healed wound was noted at a post-operative visit on day 7 in nine (0.5%) participants and on day 28 in 1574 (93.1%) participants (table 2). Most ShangRing devices had spontaneously fallen off by day 7, with 297 (17.6%) still in place during the post-operative visit (table 2).

### Adverse events

A total of 17 (1.0%) AEs were reported. Of these, nine (0.5%) were mild, five (0.3%) were moderate and three (0.2%) were severe. In five (0.3%) cases, the AE was deemed unrelated, while 12 (0.7%) was related. The most common moderate and severe AE was bleeding in two (0.1%) participants in each moderate and severe category. Most of them resolved with compression and additional vitamin K and tranexamic acid administration in four participants and led to device removal in three (table 4). The device was removed in a total of five cases secondary to AEs. There was only one serious AE related to severe bleeding, which required ring removal, ligation of the vessel causing bleed and blood transfusion (online supplemental table 2).

**Table 4:** Adverse events (AEs)

	ShangRing (n=1691)
Total number of adverse events (AE) (%)	**17** (**1.0**)
AE severity, n (%):	
Mild	**9** (**0.5**)
Bleeding	2 (0.1)
Fever	2 (0.1)
Adhesions	1 (0.1)
Poor wound healing	1 (0.1)
Insufficient skin removed	1 (0.1)
Penile oedema	1 (0.1)
Rash in thighs and genitals	1 (0.1)
Moderate	**5** (**0.3**)
Bleeding	2 (0.1)
Excessive skin removal	1 (0.1)
Infection	1 (0.1)
Painful blisters	1 (0.1)
Severe	**3** (**0.2**)
Bleeding	2 (0.1)
Insufficient skin removal	1 (0.1)
AE relatedness to circumcision	
Unrelated	5 (0.3)
Possibly related	0 (0)
Probably related	0 (0)
Definitely related	12 (0.7)
Total AEs by country, n (%)	
Kenya (n=542)	2 (0.4)
Tanzania (n=599)	4 (0.7)
Uganda (n=550)	11 (2.0)

### Parental/legally authorised representative (LAR) satisfaction

**Figure 1:**
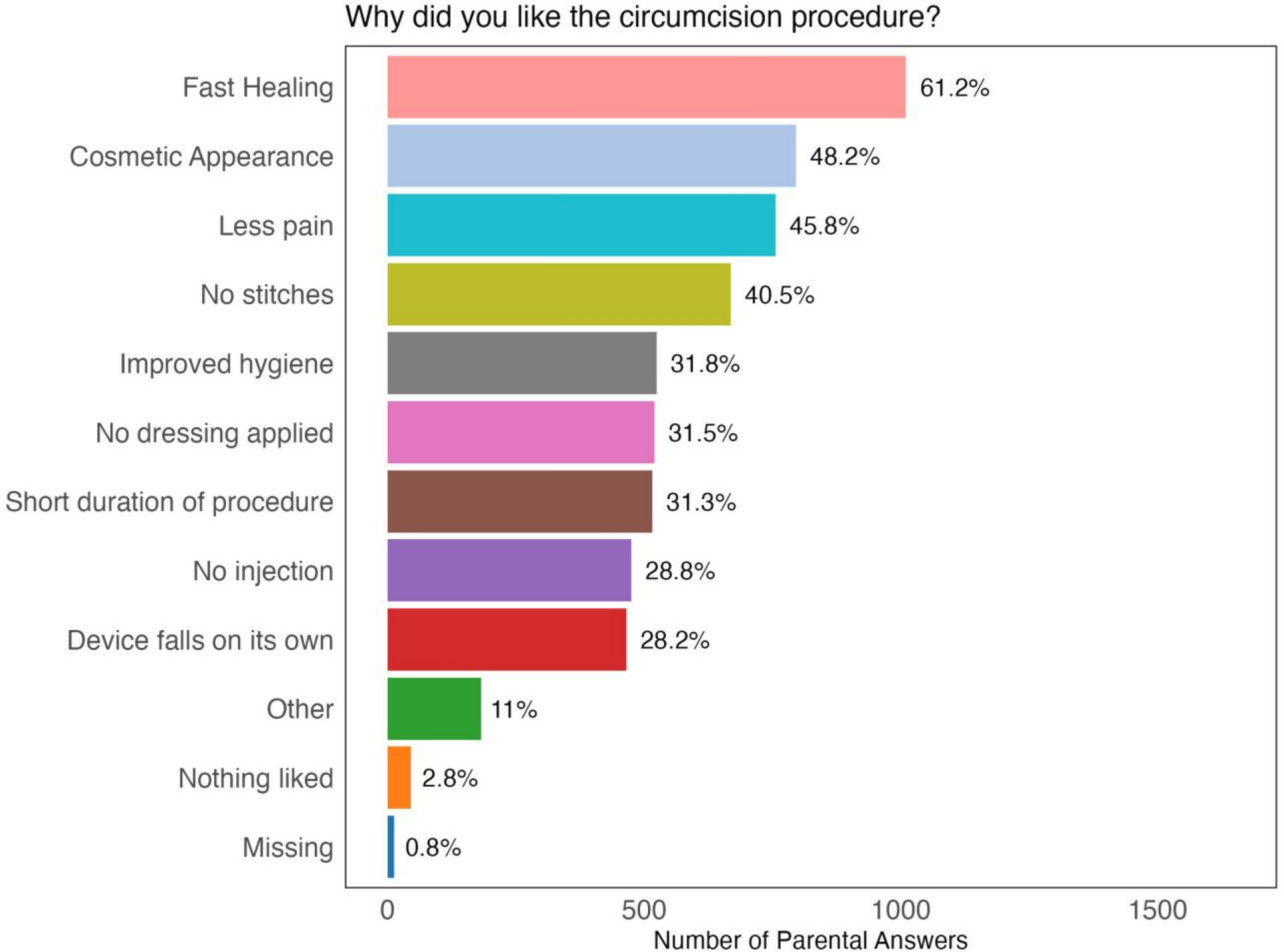
Parent/LAR satisfaction questionnaire. Answers among participants with healed penis. 40 patients were lost to follow-up and were not included. Multiple responses by parents/LAR were possible. LAR, legally authorised representative.

A total of 1650 (97.6%) parents completed a questionnaire at the 28 day follow-up, 40 (2.4%) were lost to follow-up and one with an incompletely healed penis did not complete the questionnaire. When parents were asked what they liked about the circumcision procedure with the option to choose from multiple answers, the most common answers were fast healing in 1010 (61.2%), cosmetic appearance in 796 (48.2%) and reduced pain in 756 (45.8%), no stitches in 669 (40.6%, figure 1). When asked about what they disliked about the circumcision procedure, the most common answer was nothing disliked in 1489 (90.2%), followed by more pain than expected in 84 (5.1%) and other in 42 (2.6%). A total of 1635 (99.1%) parents were satisfied with the appearance of the circumcised penis, and 1636 (99.9%) would recommend circumcision at this age to another parent.

## Discussion

In our study, we report on the clinical safety and efficacy associated with the ShangRing EIMC by midlevel medical providers in Uganda, Tanzania and Kenya. Our findings confirm its safety, with adequate wound healing, minimal AEs and efficient performance by mid-level non-physician providers, with rapid procedural time and minimal pain. Additionally, parental/LAR satisfaction rates are high.^16 17^

EIMC plays a significant role in the ongoing efforts to achieve the goal of more than 90% circumcision rates in countries with high rates of HIV transmission.^4 8^ Compared with adult/adolescent VMMC, EIMC offers early lifelong prevention, lower costs, improved wound healing and decreased AEs and complications associated with resuming sexual activity after the procedure.^19 22 23^ Despite several studies in different countries reporting on safety and efficacy outcomes from EIMC implementation, uptake remains low, for example, only 17.4% in Kenya in 2018.^23^,^26^ Field studies of devices performed by midlevel providers are essential for increasing EIMC implementation.

Compared with other circumcision devices used for EIMC, ShangRing protects the glans and urethra and has a 1% AE rate, compared with AccuCirc (2.8%) and Mogen Clamp (2.7%),^27^ including partial excision of the glans.^24 28^ The AccuCirc device also has a 3.1% device malfunction rate, with incomplete resection of the foreskin, requiring providers to complete the cut with sterile scissors, which in some cases resulted in prolonged bleeding and needing sutures.^24 29^ There were only two complications associated with wound healing, one cosmetic in nature, which was deemed secondary to poor hygiene, and the other due to a surgical site infection. Urethral fistulas are an uncommon but morbid complication that may result from freehand circumcision and with the use of devices, where the retained device can migrate proximally, resulting in fistula creation.^27 30^ Most importantly, we did not identify any of the serious penile injuries reported with other devices, such as penile amputation with the use of the Mogen Clamp or urinary fistula with the Plastibell device.^27 28^ Moreover, we do not anticipate an increased risk of bleeding complications in infants born at home, as the double-ring mechanism ensures effective haemostasis, reducing the likelihood of excessive bleeding even in the absence of vitamin K.

Rapid wound healing was the most common reason parents gave as to why they liked the procedure, supporting the importance of ensuring adequate wound healing to maintain parental approval and cultural acceptance.^31^ Past studies in adults using ShangRing reported slower healing (87.2% by 35 to 42 days postcircumcision), highlighting a neonatal advantage.^32^

Our results support trained mid-level medical providers can successfully and safely perform ShangRing EIMC. We believe that infants with phalluses that seem too small for the smallest size ShangRing (Size Z, inner ring diameter of 11 mm) might need to wait until the phallus has grown to a larger size or perhaps use an alternate device if available, such as Gomco clamp.^33^ Furthermore, similar to an AccuCirc field study, there have been no reported differences between physicians and non-physician providers for the ShangRing EIMC in AEs related to circumcision.^29 34^ Finally, non-physician providers are already trained to perform dorsal slits as part of the standard VMMC programme, eliminating the need for additional training, equipment or costs. This underlines the safety of task-shifting EIMC to midlevel providers using the ShangRing.

Pain remains a common barrier to EIMC uptake.^12 24^ However, neonatal circumcision is associated with less pain, given a less vascularised and thickened foreskin.^23 35^ In our study, only 2.6% of cases required supplemental local injectable anaesthesia, and the dwell time was similar to other devices.^16^ Although NIPS has not been designed for the assessment of pain associated with a procedure,^21^ it nonetheless represents a non-biased validated measure of acute pain and was thus used in our previously reported randomised controlled trial, which showed low pain levels as reported by NIPS and no difference in pain when compared with EIMC using the Mogen clamp.^16^ Compared with previous field studies in other devices, where local injectable anaesthesia was used,^24^ our study further demonstrates the safety and efficacy of performing these procedures with topical anaesthesia, as recommended by the WHO for EIMC, which may help to achieve a greater uptake by parents/LARs.^19^

Acceptability of EIMC by the community represents a critical component to achieving high rates of circumcision and, thus, maintaining a sustainable uptake.^12^ Potential barriers include safety, pain and cultural considerations.^12 36 37^ In our study, we investigated parental satisfaction as a recognised important tool leading to the potential to scale up. Although previous field studies of MC in adolescents and adults have explored parental/LAR satisfaction and reported similar rates of 99%, to our knowledge, this is the first study to investigate the reason for high satisfaction further.^29^ Protection against HIV and improved hygiene has been reported as the main reasons why parents/LAR are willing to accept circumcision, which compares to our study.^24 29^

Family dynamics play a role in decision-making.^38 39^ As Bailey *et al* reported in an AccuCirc field study in Kenya, when mothers were asked who should participate in the decision, 82.9% reported both father and mother were important, 8.1% reported the mother, 7.9% the father, while only 1.3% thought the healthcare proxy was the most important one.^24^ However, Mavhu *et al* reported that in Zimbabwe, the father makes the final decision most of the time, although the mother could participate in decision-making.^38^ Importantly, both studies have shown a greater acceptance of EIMC from circumcised fathers, and as this population grows with increasing VMMC efforts, so hopefully will EIMC acceptance. Targeting all decision-makers and community healthcare providers is key to scaling up VMMC.

Some of the limitations to be considered are the inherent bias associated with a single-arm, non-blinded study. Additionally, heterogeneity is related to different study sites, personnel and providers. However, this is also one of the study’s strengths’ as it improves our results’ generalisability in the intended settings. Lastly, only participants who are born at hospitals or clinics with access to trained healthcare providers were screened to participate, which is not representative of all neonates in sub-Saharan Africa. Future efforts involve incorporating other healthcare workers who provide general neonatal care to counsel, perform and provide follow-up care after the procedure. This would entail generating a more comprehensive spread of knowledge of the procedure and expectation and knowing when to refer to physicians or clinical officers.

## Conclusions

This clinical trial is the first field study of the ShangRing for EIMC, which reports satisfactory safety and efficacy when performed by non-physician providers, with minimal AEs and high parental/LAR satisfaction. This study supports the notion of moving forward with broader implementation of the ShangRing for EIMC. Future efforts should be aimed at increasing access to ShangRing EIMC and thus up-scaling MC in African countries with high rates of HIV.

## Supplementary material

10.1136/bmjgh-2024-017903 online supplemental file 1

## Data Availability

Data are available upon reasonable request.
