# Supplementary material for: Safety and efficacy of the ShangRing for early infant male circumcision in the routine clinical setting
Source: BMJ Glob Health. 2025 Sep 25;10(9):e017903. doi: 10.1136/bmjgh-2024-017903 (PMC12481264; doi:10.1136/bmjgh-2024-017903)
Supplement: online supplemental file 1 [file bmjgh-10-9-s001.docx]

**Supplementary Table 1. Description of problems or difficulties with the circumcision procedure**

| **Participant number(s)** | **Country** | **Description** |
| --- | --- | --- |
| 2HB011 | Kenya | Difficulty in inserting the ring due to penis size |
| 2HB026 | Kenya | Inserting the ring took too long, the penis was too small |
| 2IR087 | Tanzania | It was difficult to remove adhesion |
| 2HB050 | Kenya | The penis was too small |
| 2HB070 | Kenya | The penis was too small, that made ring placement to take long |
| 2HB051 | Kenya | The penis was too small. |

**Supplementary Table 2. Detailed description of adverse events.**

| AE | Country | AE severity | AE description | AE relatedness | AE device action |
| --- | --- | --- | --- | --- | --- |
| 1 | Kenya | Moderate | Bleeding underneath the shangring | Related | Device removed |
| 2 | Kenya | Moderate | Infected wound | Related | Device removed |
| 3 | Tanzania | Severe | Insufficient foreskin removal | Related |  |
| 4 | Tanzania | Mild | A whitish fat- like layer covered on the circumcised wound and glans of penis due to poor hygiene and improper application of the vaseline gel. | Related | Device removed |
| 5 | Tanzania | Moderate | Bleeding which stops with compression | Related | Device not removed |
| 6 | Tanzania | Moderate | Excessive skin removal to the left side of the shaft up to base of the penis | Related |  |
| 7 | Uganda | Severe | Bleeding | Related | Device removed |
| 8 | Uganda | Mild | Febrile with temperature of 39C | Unrelated | Device not removed |
| 9 | Uganda | Mild | Rash in between thighs and genitalia | Unrelated |  |
| 10 | Uganda | Mild | Bleeding | Related |  |
| 11 | Uganda | Mild | Febrile with temperature of 38.2C | Unrelated |  |
| 12 | Uganda | Severe | Bleeding from the ring | Related | Device removed |
| 13 | Uganda | Moderate | Painful blisters around abdomen, pelvis and legs on the anterior side of the body | Unrelated |  |
| 14 | Uganda | Mild | Bleeding at the frenulum | Related | Device not removed |
| 15 | Uganda | Mild | Insufficient skin removal | Related |  |
| 16 | Uganda | Mild | Small adhesion | Related |  |
| 17 | Uganda | Mild | Mild swelling below the ring | Related | Device not removed |
